# Physicochemical and Perceived Olfactory Changes in Black Soldier Fly (*Hermetia illucens*) Larvae Oil Under Domestic Cooking Temperatures

**DOI:** 10.3390/foods14132333

**Published:** 2025-06-30

**Authors:** Kian Aun Chang, Sze Ying Leong, Lye Yee Chew, Ching Qi Lim, Meng Jack Lim, Zongwei Ong, Sook Wah Chan

**Affiliations:** 1 School of Biosciences, Faculty of Health and Medical Sciences, Taylor’s University, Subang Jaya 47500, Selangor Darul Ehsan, Malaysia; changkianaun@gmail.com (K.A.C.); lyeyee.chew@taylors.edu.my (L.Y.C.); sookwah.chan@taylors.edu.my (S.W.C.); 2 Food Security and Nutrition Impact Lab, Taylor’s University, Subang Jaya 47500, Selangor Darul Ehsan, Malaysia

**Keywords:** food waste, circular economy, food security, insect larvae, insect oil quality, odour perception, fatty acid composition

## Abstract

The rapid growth and sustainable production of black soldier fly larvae (BSFL) contribute positively to the circular economy. This study profiled the fatty acid composition of crude BSFL oil, followed by an evaluation of its physicochemical properties under domestic cooking temperatures (up to 180 °C, 30 min). Odour evaluation of the BSFL oil was also performed using 10 trained panellists for attributes such as fishy, nutty, oily, meaty/savoury, roasted, and pungent. The results indicated that BSFL oil contains palmitic (23.69%), oleic (30.90%), and linoleic (21.81%) acids in relatively similar proportions, representing a mix of saturated, monounsaturated, and polyunsaturated fatty acids. Heating caused BSFL oil to be darker and more viscous. The peroxide and free fatty acid values also increased significantly (*p* < 0.05) with rising temperatures, indicating limited oxidative stability and reduced suitability of BSFL oil for cooking purposes. The perceived intensity of odour attributes, particularly fishy and oily notes, increased concomitantly with higher cooking temperatures. Refining processes and antioxidants may assist in improving the thermal stability of BSFL oil for culinary applications.

## 1. Introduction

As global demand for sustainable and alternative food and bio-based products continues to grow, black soldier fly larvae (BSFL) oil has emerged as a promising candidate for the food industry due to its nutritional profile and environmentally friendly production. Extracted from the larvae of *Hermetia illucens*, BSFL oil is rich in fatty acids and bioactive compounds [1], positioning it as a promising alternative to conventional cooking oils. Unlike tropical cooking oils such as palm and coconut oil, BSFL oil contains a diverse mix of saturated and unsaturated fatty acids, which may offer distinct nutritional and functional properties [1]. Moreover, BSFL oil offers the advantage of being derived from larvae that convert organic residue into valuable nutrients, making its production more sustainable and resource efficient [2]. However, before BSFL oil can be widely adopted commercially, it is essential to understand its stability, quality, and performance under common culinary conditions.

Refined vegetable oils typically exhibit a lighter colour, a neutral to bland flavour profile, an extended shelf life, and a higher smoke point compared with their crude counterparts [3]. These differences are largely attributed to the removal of oxidation-prone compounds such as free fatty acids, phospholipids, pigments, and other impurities through refining processes consisting of degumming, neutralisation, bleaching, and deodourisation [3,4]. As a result, refined oils tend to demonstrate greater thermal stability, slower degradation, and reduced off-flavour development during heating. While Mai et al. [5] have reported on the physicochemical changes in BSFL oil following purification, highlighting improved stability, studies exploring its culinary applications under thermal conditions remain limited. Domestic cooking processes requiring the use of cooking oils include deep frying, pan frying, stir frying, baking, roasting, grilling, *sautéing*, and *confit*, with temperatures ranging from 60 °C to 200 °C [4,6,7]. Heating oil at high temperatures will result in physical, chemical, and sensory transformations that can affect oil quality, nutritional value, and consumer appeal [6]. High temperatures can lead to the oxidation, hydrolysis, and polymerisation of oils, producing compounds such as trans fats, aldehydes, and free radicals that may be harmful to human health [4]. These changes are well-documented for edible vegetable oils [6,8,9], but little is known about how alternative oils such as BSFL oil behave in such high-temperature conditions. This gap in knowledge presents a barrier to the broader application of BSFL oil as a viable substitute for conventional oils in cooking.

From a sensory perspective, the odour of BSFL oil may be perceived as undesirable by consumers, presenting a challenge for its incorporation into food products and nutraceuticals. Notably, the use of BSFL oil in high-temperature cooking applications raises concerns about potential changes in its odour profile due to heat exposure. Different oils have been shown to generate varying concentrations of aldehydes, which contribute to flavour and aroma, influenced by their fatty acid composition and oxidation rates [10]. Studies by Huseynli et al. [2] and Lee et al. [11] have explored the volatile compounds present in oils derived from various insect larvae (*T. molitor*, *A. dichotoma*, *P. brevitarsis seulensis*, *H. illucens*) using gas chromatography-mass spectrometry. Nonetheless, there is a scarcity of research quantifying the mean intensity of specific odour attributes associated with BSFL oil using human subjects, particularly when subjected to heat.

This study aims to fill the identified gaps by investigating the fatty acid composition, followed by the physical, chemical, and perceived olfactory changes that occur in BSFL oil under domestic cooking temperatures. It shall focus on key parameters such as colour, viscosity, peroxide value, iodine value, and free fatty acid level to assess the thermal stability and oxidation behaviour of BSFL oil. By comprehensively examining these properties, the current study seeks to provide critical insights into the performance and acceptability of BSFL oil for commercial use. Understanding these changes is crucial for evaluating the potential of BSFL oil as a sustainable alternative in high-temperature cooking processes, ensuring it meets the necessary standards for quality, stability, and market acceptance standards in the food industry.

## 2. Materials and Methods

### 2.1. Chemicals and Reagents

Acetic acid (CAS No. 64-19-7), sodium thiosulphate pentahydrate (CAS No. 10102-17-7), and potassium dichromate (CAS No. 7778-50-9) were obtained from Classic Chemicals Sdn. Bhd. (Shah Alam, Selangor, Malaysia). Wijs reagent (CAS No. 7790-99-0), sodium hydroxide (CAS No. 1310-73-2), hexane (CAS No. 110-54-3), and methanol (CAS No. 67-56-1) were purchased from Chemiz (M) Sdn. Bhd. (Shah Alam, Selangor, Malaysia). Isooctane (CAS No. 540-84-1) and isopropanol (CAS No. 67-63-0) were sourced from J.T. Baker (Radnor, PA, USA). Hydrochloric acid (CAS No. 7647-01-0) and potassium hydroxide (CAS No. 1310-58-3) were supplied by Fisher Scientific (M) Sdn. Bhd. (Selangor, Malaysia). Phenolphthalein (CAS No. 77-09-8) was acquired from Sigma-Aldrich (M) Sdn. Bhd. (Petaling Jaya, Selangor, Malaysia), potassium iodide (CAS No. 7681-11-0) from HmbG Chemicals (Hamburg, Germany), soluble starch (CAS No. 9005-84-9) from R&M Chemicals (Semenyih, Selangor, Malaysia), and cyclohexane (CAS No. 110-82-7) from Macron Fine Chemicals (Radnor, PA, USA). Fatty acid methyl ester reference was sourced from Merck KGaA (Supelco^®^, CRM47885, Darmstadt, Germany). All chemicals used in this study were of analytical grade. Deionised water (Elga Micromeg Deioniser, High Wycombe, UK) was used for the preparation of all chemical solutions.

### 2.2. Reference Cooking Oils

Commercially available refined, bleached, and deodourised coconut cooking oil (Medella^®^, Bountiful Ventures Sdn. Bhd., Subang Jaya, Selangor, Malaysia; packed in opaque high-density polyethylene bottles) and palm olein (Buruh, Lam Soon Edible Oils Sdn. Bhd., Shah Alam, Selangor, Malaysia; packed in transparent polyethylene terephthalate bottles) were purchased from a supermarket in Selangor, Malaysia.

### 2.3. Black Soldier Fly Larvae Rearing and Oil Extraction

**Figure 1:**
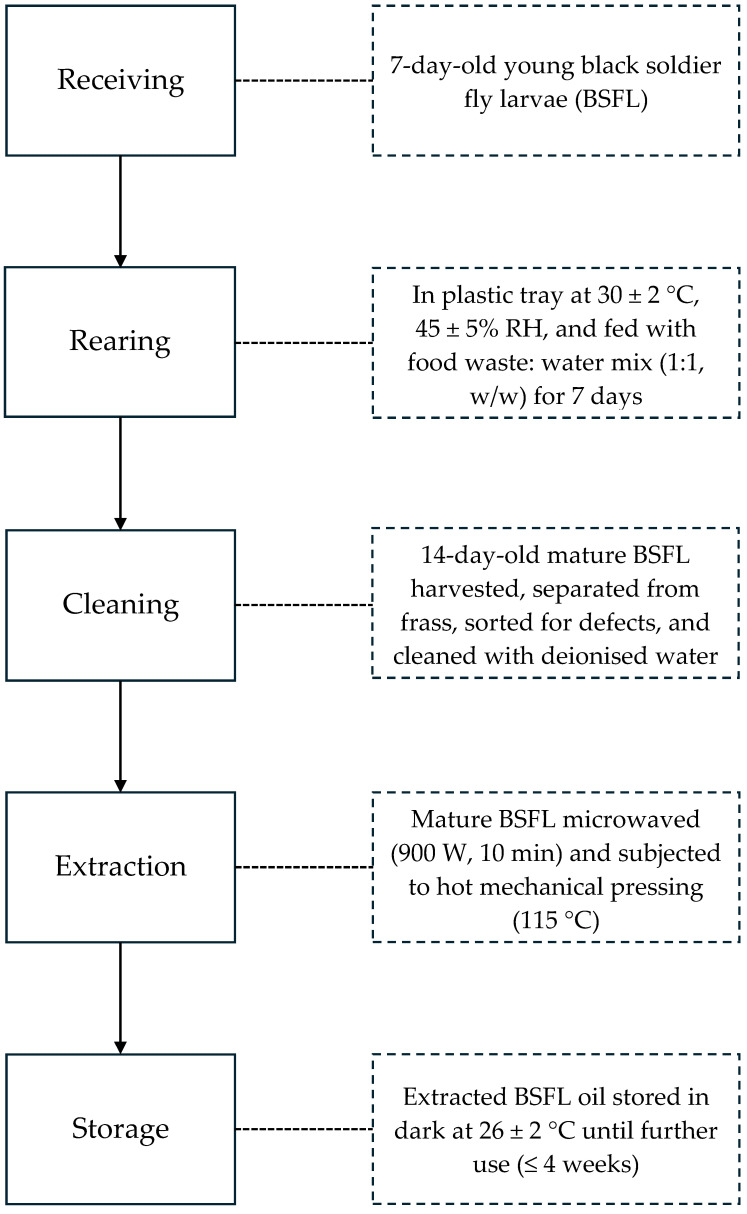
Flowchart of BSFL rearing and oil extraction. BSFL denotes black soldier fly larvae. RH denotes relative humidity.

Figure 1 illustrates the flowchart of BSFL rearing and oil extraction, which were performed according to the work of Saviane et al. [12] with minor modifications. Seven-day-old black soldier fly (*Hermetia illucens*) young larvae were provided by Entomal Biotech Sdn. Bhd. (Kuala Lumpur, Malaysia) Supplementary Data S1 and Figure S1). These young larvae were reared for an additional seven days in experimental trays, with approximately 150 g of larvae per tray in a controlled environment of 30 ± 2 °C and 45 ± 5% relative humidity. They were fed a diet of 15 kg plant-based food residues, comprising a mixture of food waste (ca. 60% bread and ca. 40% vegetables, soy-based ingredients, and fruits) homogenised with water at a 1:1 (*w*/*w*) ratio. At day seven, the larvae were harvested, separated from the frass, sorted for defects, rinsed with deionised water, and subsequently microwaved (Sharp, R639ES, China) at 900 W for 10 min to prepare them for oil extraction. Mechanical hot pressing extraction was performed using a customised oil press machine operating at 115 °C. This extraction method was selected, as it represents a simple and safe technology for producing high-quality oil with no chemical residue [13,14]. The extracted crude BSFL oil was then stored under dark conditions at room temperature (26 ± 2 °C) to mimic typical household storage temperature of cooking oils in tropical climates such as Malaysia. Samples were stored for no longer than four weeks prior to physicochemical analyses and sensory odour profiling.

### 2.4. Exposure of BSFL Oil to Domestic Cooking Conditions

Initially, the extracted BSFL oil was pre-heated in a water bath (Memmert, WNB 14, Schwabach, Germany) at 36 °C. This step was necessary to melt any fat crystals that formed during post-extraction storage. The oil was then thoroughly mixed and filtered through two layers of cheesecloth to remove any contaminants. The domestic cooking temperatures were applied at low (120 °C) and high (180 °C) ranges using a convection oven (Memmert, UF 110, Schwabach, Germany), following the methods of Mitrea et al. [6] and Zhuang et al. [15] with minor modifications. For each cooking temperature, the oven was preheated for at least 1 h. Then, 50 mL of the oil sample was transferred to a 150 mL white porcelain ramekin, covered with aluminium foil, and heated in the convection oven for 30 min at 100% fan speed. After heating, the oil samples were allowed to cool to room temperature for another 30 min before physicochemical analyses. BSFL oil samples equilibrated at 36 °C for 30 min were considered unheated control samples. Similar domestic cooking conditions were applied to both coconut oil and palm olein, with their respective unheated counterparts serving as control samples. These oils were chosen because they are often utilised in households and restaurants worldwide for thermal food processing, such as frying [6]. Additionally, it has been reported that the fatty acid profile of BSFL oil is comparable to that of coconut and palm oil [16]. The domestic cooking conditions were replicated to prepare BSFL oil samples for sensory odour profiling (see Section 2.6), aimed at generating odour descriptors and training for evaluating the perceived intensity of specific odour attributes. All oil samples were stored in the dark at room temperature until further use.

### 2.5. Physicochemical Analyses

Fatty acids in all the oil samples (BSFL oil, coconut oil and palm olein) were converted to fatty acid methyl esters (FAME) following the International Union of Pure and Applied Chemistry (IUPAC) Commission on Oils, Fats, and Derivatives Test Method 2.301:1987 with minor modifications before gas chromatography-mass spectrometry analysis [17]. The standard used to determine the fatty acid composition was Supelco 37 Component FAME Mix (Merck KGaA, CRM47885, Darmstadt, Germany). The fatty acid composition was expressed as the percentage of the peak area for each compound relative to the total peak area. Colour and viscosity were analysed using a pre-calibrated benchtop spectrophotometer (HunterLab, ColorFlex EZ, Murnau, Germany) and a viscometer (Brookfield, DV2T, Middleboro, MA, USA), respectively. Peroxide value, iodine value, and free fatty acid value were determined titrimetrically using Malaysian Palm Oil Board (MPOB) test methods MBOT p2.3:2004, MBOT p3.2:2004, and MPOB p2.5:2004, respectively, with minor modifications [18,19]. The elaborated steps for physicochemical analyses conducted in the present study are detailed in Supplementary Data S2–S6.

### 2.6. Odour Characterisation

Ten trained panellists (seven males, three females), aged 20 to 22 years, were selected from an initial pool of 21 participants aged 20 to 30 years, who were recruited via convenience sampling at Taylor’s University Lakeside Campus, Selangor, Malaysia. All candidates were screened for olfactory acuity, food neophobia, and ability to complete sensory evaluation tasks. Only panellists who met all criteria were included in the odour evaluation of three BSFL oil samples before (control/unheated) and after heating to domestic cooking temperatures (120 °C and 180 °C). Each panellist underwent training on odour descriptor development (Supplementary Table S1) and scale used. The panel received a total of 18 h (9 × 2 h sessions) of product-specific training before the odour evaluation. Sensory evaluation took place in individual testing booths at Taylor’s University Food Science Lab under controlled lighting conditions. Each panellist evaluated the three BSFL oil samples (5 mL each, allowing volatiles to equilibrate in the headspace of 30 mL amber bottles with metal caps for at least 1 h at a room temperature of 26 ± 2 °C) in a randomised, balanced design over two independent sessions, with each sample presented twice per session. For each sample, panellists rated the perceived intensity of six agreed-upon odour attributes (fishy, nutty, oily, meaty/savoury, roasted, and pungent; Supplementary Table S2) on an unstructured 150 mm line scale anchored at 10 mm with “None” and 140 mm with “Intense” Supplementary Data S7.5). Sensory data collection was conducted with EyeQuestion (Logic8 B.V., Gelderland, The Netherlands). Between samples, panellists were provided with roasted coffee beans to neutralise their sense of smell and instructed to take a 1 min break to avoid olfactory fatigue. Ethical approval for the trained sensory panel evaluating BSFL oil was obtained from Taylor’s University Human Ethics Committee (Reference number: HEC 2023/273). Further details about the odour evaluation setup are presented in Supplementary Data S7.

### 2.7. Statistical Analyses

All physicochemical analyses were conducted in triplicate and reported as mean ± standard deviation. Data collected from the physicochemical and sensory odour analyses were evaluated using Statistical Package for Social Sciences (SPSS) (IBM Corporation, Version 25, Armonk, New York, USA). A one-way analysis of variance (ANOVA) was employed to assess the significant differences, at *p* < 0.05, in the physicochemical changes between the oil samples exposed to different temperatures, followed by Tukey’s honest significant difference (HSD) test. The results from the odour evaluation of BSFL oil were evaluated using ANOVA with a generalised linear model (GLM). The main effects (oil sample, panellist, evaluation replication) and their 2-way interactions (panellist × replication, panellist × sample, replication × sample) were evaluated, with panellist ratings as dependent variables. Levene’s test was used to confirm homogeneity of variance (*p* > 0.05). Tukey’s HSD test was used to identify significant differences (*p* < 0.05) among the perceived odour intensity between the BSFL oil samples.

## 3. Results and Discussion

### 3.1. Fatty Acid Composition of Oils Prior to Heating

Cooking oils high in saturated fatty acids (SAFA) and monounsaturated fatty acids (MUFA) generally demonstrate greater oxidative stability at elevated temperatures, whilst those rich in polyunsaturated fatty acids (PUFA) are more susceptible to oxidation [20]. Therefore, the fatty acid composition of cooking oils is a key factor influencing their stability during domestic cooking. Table 1 presents the fatty acid composition of BSFL oil, coconut oil, and palm olein, while Supplementary Figure S2 displays their GC-MS chromatograms along with the corresponding fatty acid identifications. In general, BSFL oil exhibited the highest content of PUFA at ca. 22%, whilst coconut oil and palm olein contained the highest content of SAFA and MUFA at ca. 83% and 45%, respectively (Supplementary Figure S3 and Table S4).

Amongst the experimented oils, BSFL oil demonstrated a relatively balanced fatty acid profile with 43.22% SAFA, 34.96% MUFA, and 21.81% PUFA (SAFA:MUFA:PUFA; 2.0:1.6:1.0). Though the fatty acid profile reported in the current study is reflective of crude BSFL oil, the fatty acid composition of crude and refined BSFL oil is comparatively similar, according to the study of Mai et al. [5]. It is crucial to note that the fatty acid composition of BSFL oil can be highly dependent on the feeding substrate; hence, the data presented in this study are specifically relevant to the rearing conditions applied, which involved plant-based food residues consisting of a homogenised mixture of ca. 60% bread and ca. 40% vegetables, soy-based ingredients, and fruits. This consistent substrate formulation was intentionally used in the current study to ensure uniformity in the diet of BSFL, which was essential for conducting a controlled sensory evaluation of the resulting oils. Whilst it is indeed important to investigate how different feeding substrates may influence the quality of BSFL oil, this aspect falls beyond the scope of the current study, which primarily focuses on examining the thermal stability behaviour of BSFL oil. To strengthen the interpretability and reproducibility of BSFL oil characterisation in future research, precise quantification of individual feed components should be prioritised.

Coconut oil contained the highest levels of SAFA (82.70%) contributed by lauric acid (C12:0, 24.88%) and myristic acid (C14:0, 18.77%). This implies that coconut oil is highly resistant to oxidation due to the absence of double bonds [4]. The medium-chain triglyceride (MCT) found in virgin coconut oil was reported to have good oxidative stability when heated up to 250 °C for 1 h [21]. On the other hand, palm olein and BSFL oil showed comparable SAFA levels, contributed primarily by palmitic acid at 30.24% and 23.69%, respectively. Concurrently, their total unsaturated fatty acid (TUFA) levels (palm olein, 60.24%; BSFL oil, 56.77%), comprising MUFA (palm olein, 45.02%; BSFL oil, 34.96%) and PUFA (palm olein, 15.22%; BSFL oil, 21.81%), were also comparable. Altogether, it can be implied that palm olein and presumably BSFL oil demonstrate superior oxidative stability compared with coconut oil, which may be attributed to their balanced proportion of saturated and monounsaturated fatty acids. This composition enhances thermal stability, as saturated fatty acids resist oxidation, while monounsaturated fatty acids are less susceptible to oxidative degradation than polyunsaturated fatty acids [4]. Furthermore, unlike MCT, oils having longer-chain fatty acids contribute to higher smoke points [22], whilst oils rich in MUFA, such as palm olein, are less susceptible to thermal degradation and oxidation [8].

### 3.2. Physicochemical Properties of Oils Prior to Heating

The colour, viscosity, peroxide value (PV), iodine value (IV), and free fatty acid (FFA) value of BSFL oil, coconut oil, and palm olein are presented in Table 2 (and summarised in Supplementary Table S4).

BSFL oil was found to be significantly (*p* < 0.05) darker, redder, and yellower than coconut oil and palm olein. This could be due to the presence of colour compounds and natural pigments inherent to crude oils [5]. It is important to note that oils with a darker colour and higher redness and yellowness may limit their use in food applications, as they can darken the appearance of final products. Additional refining processes (e.g., degumming, neutralisation, bleaching, and deodourisation) are recommended to address this limitation for BSFL oil. On the other hand, the viscosity of oil is crucial for mouthfeel as it affects the rate of oil draining from cooked or fried food and consequently, the oil content and taste sensation obtained [23]. In the present study, the viscosity of BSFL oil was significantly higher (*p* < 0.05) than coconut oil but comparable with palm olein (*p* > 0.05) (Table 1), which could be justified by the differences in their fatty acid composition [23]. BSFL oil and palm olein contain a higher proportion of long-chain fatty acids such as palmitic (C16:0) and oleic (C18:1) acids. These fatty acids have stronger intermolecular interactions due to longer carbon chains (i.e., higher molecular weight), hence contributing to higher viscosity [23]. Besides, BSFL oil in its crude form contains phospholipids, waxes, and other impurities that could increase its viscosity. In contrast, coconut oil is primarily comprised of medium-chain fatty acids such as lauric (C12:0) and myristic (C14:0) acids. These fatty acids have shorter carbon chains (i.e., lower molecular weight), resulting in weaker intermolecular forces and, hence, lower viscosity.

The PV is used to indicate early stages of oxidation in oil by measuring primary oxidation products such as hydroperoxides. BSFL oil (2.62 meq/kg oil) showed an intermediate PV between coconut oil (0.78 meq/kg oil) and palm olein (8.38 meq/kg oil), suggesting moderate oxidative stability that was significantly different (*p* < 0.05) from vegetable oils. From a regulatory perspective, the PV value of all oils fulfilled the Codex Alimentarius [24] specification of ≤10.0 meq/kg oil (Supplementary Table S3). Nonetheless, the higher PV of palm olein, when compared with BSFL oil (stored in dark conditions) and coconut oil (which comes in opaque bottles), could be attributed to its transparent primary packaging material. Transparent packaging allows oil to be exposed to light when displayed on the shelf, subsequently promoting photo-oxidation and free radical formations. In Malaysia, it is common for commercial palm-based cooking oil to be packed in transparent/translucent plastic pouches and polyethylene terephthalate (PET) bottles [25].

Since IV is an indicator of the degree of unsaturation in oil, the IV of BSFL (52.08 g I_2_/100 g oil) was significantly higher (*p* < 0.05) than that of coconut oil (12.02 g I_2_/100 g oil) but lower than palm olein (63.31 g I_2_/100 g oil). These observations align with Table 1, where the balanced fatty acid composition of BSFL oil results in intermediate oxidative stability (i.e., better than palm olein but not as stable as coconut oil), whilst the low IV value of coconut oil reflects its high SAFA content (i.e., high degree of saturation/double bonds). The IV of crude BSFL oil in this study is similar to that reported by Mai et al. [5] for refined BSFL oil at 53.7 g I_2_/100 g oil. The FFA value measures the level of hydrolysed fatty acid in oil. According to the Malaysian standard, the FFA of refined coconut oil (MS 239:1987; [26]) and palm olein (MS 816:2007; [27]) were set at ≤0.10%. A higher FFA level indicates more oil degradation, often resulting in undesirable off-flavour and rancidity. The FFA content of coconut oil and palm olein was comparable at 0.13% (but slightly higher than the Malaysian standard), whereas the FFA of BSFL oil was nearly nine-fold higher at 1.13%. It could be that the hot mechanical pressing used for BSFL oil extraction, without further refining, has led to higher FFA levels due to incomplete removal of enzymes and moisture that promote hydrolysis [23]. This further supports the importance of refining crude BSFL oil to attain regulatory compliance (e.g., ≤0.10% FFA) and to ensure its suitability for direct consumption and consumer safety. Notably, conventional oil refining processes are capable of reducing FFA levels to below 0.5%, depending on the oil and refining method used [4].

### 3.3. Physicochemical Properties of Oils After Heating

**Figure 2:**
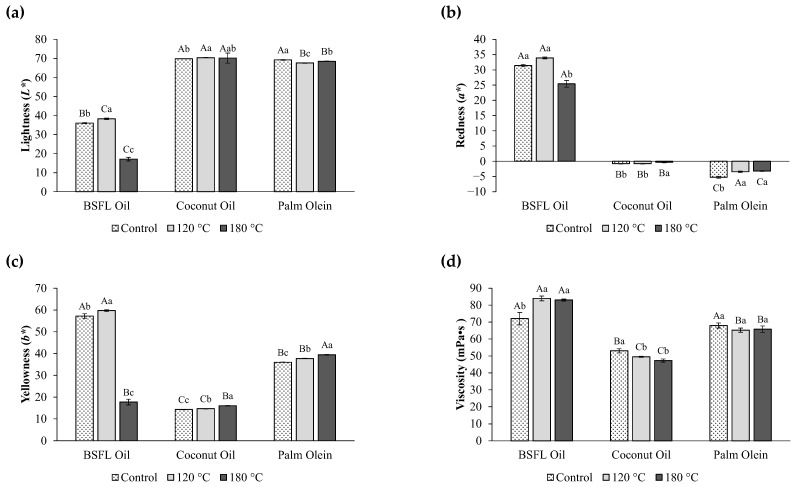
Physical properties of BSFL oil, coconut oil, and palm olein before (referred to as “Control”) and after heating: (**a**) lightness, (**b**) redness, (**c**) yellowness, and (**d**) viscosity. Values are mean ± standard deviation (*n* = 3). Bars with different upper-case letters indicate statistically significant differences (*p* < 0.05) between the different oils at the same temperature. Bars with different lower-case letters indicate statistically significant differences (*p* < 0.05) between the different temperatures within the same type of oil.

Colour plays an essential role in the visual assessment of oil quality, as it depicts changes due to production and storage conditions, and significantly influences consumer preferences, with most opting for a consistent golden colour of the oil and food products, especially after frying [28]. When comparing L* values for different oil types across the same temperature, at either 120 °C or 180 °C, there was a significant difference (*p* < 0.05) between them, with BSFL oil being the darkest (i.e., lowest L*), especially at 180 °C (Figure 2a and Supplementary Table S5). Further, a comparison of colour indexes for the same oil type across different temperatures disclosed a relatively stable trend for coconut oil and palm oil, with only slight changes to the L*, a*, and b* values at either 120 °C or 180 °C (Figure 2a–c and Supplementary Table S6), indicating minor shifts toward darker, reddish, and yellowish colours. For BSFL oil, heating at 120 °C was sufficient to significantly increase (*p* < 0.05) the L* and b* values when compared with the control, whilst a higher temperature of 180 °C was required to lower its redness index. The colour variation of BSFL oil in response to increasing temperature exposure is illustrated in Supplementary Figure S4. The changes in oil colour upon heat exposure can be attributed to oxidation and polymerisation reactions of unsaturated fatty acids. Polymerised fat could lead to gum formation, foam formation, colour darkening, and further deterioration of frying oil [4]. Moreover, the present study observed a marked reduction in the colour indexes of BSFL oil at 180 °C, signalling its poor heat stability at elevated temperatures, especially beyond 120 °C. This necessitates additional refining processes to improve the stability and colour consistency of BSFL oil over higher temperatures and repeated heating cycles in order to support its broader application in food systems.

The viscosity of palm olein remained stable and statistically insignificant (*p* > 0.05) before and after the application of domestic cooking temperatures (Figure 2d). This suggests the resilience of palm olein toward high-temperature conditions. Coconut oil, on the other hand, showed a decrease in viscosity when exposed to heat, and its viscosity at 120 °C and 180 °C was similar (*p* > 0.05). This is likely due to its high SAFA content, which solidifies at low temperatures (<20 °C) and liquefies with increased temperature [6]. Conversely, BSFL oil experienced a significant increase (*p* < 0.05) in viscosity at 120 °C and 180 °C, likely due to oxidation and polymerisation reactions during heat exposure [4]. These reactions result in the formation of high molecular weight compounds and cross-linked structures that elevate viscosity. Clearly, these findings imply that the physical properties of BSFL oil undergo substantial changes, particularly at 180 °C.

**Figure 3:**
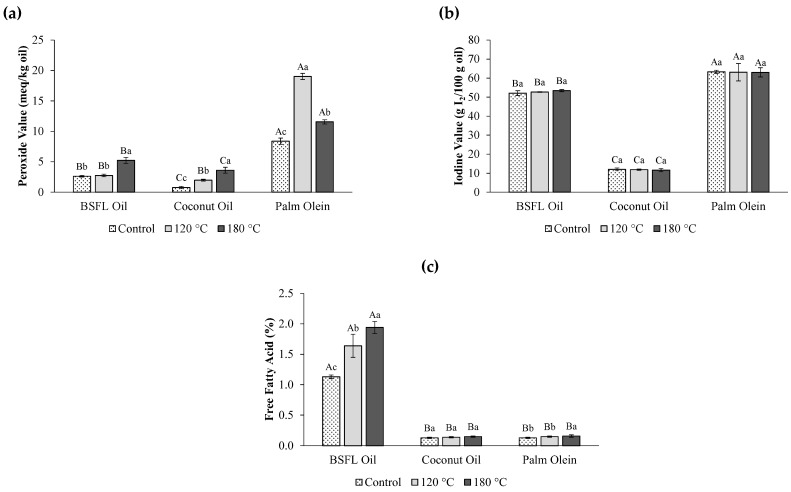
Chemical properties of BSFL oil, coconut oil, and palm olein before (referred to as “Control”) and after heating: (**a**) peroxide value, (**b**) iodine value, and (**c**) free fatty acid value. Values are mean ± standard deviation (*n* = 3). Bars with different upper-case letters indicate statistically significant differences (*p* < 0.05) between the different oils at the same temperature. Bars with different lower-case letters indicate statistically significant differences (*p* < 0.05) between the different temperatures within the same type of oil.

Oils with a higher PV indicate they have undergone oxidative damage. Theoretically, oil rich in SAFA, MUFA, and PUFA is expected to have low, moderate, and high peroxide formation, respectively. All experimental oils showed an increase in PV after being subjected to heat (Figure 3a). Notably, BSFL oil displayed a significant increase (*p* < 0.05) in PV at 180 °C when compared with the control, whilst coconut oil and palm olein started exhibiting a significant increase (*p* < 0.05) at 120 °C. Unlike BSFL oil and coconut oil, which demonstrated a gradual and linear increase in PV as the cooking temperature rose, the PV of palm olein showed, firstly, a sharp increase from 8.38 meq/kg oil (unheated/control) to 19.02 meq/kg oil at 120 °C, followed by a decrease to 11.56 meq/kg oil at 180 °C. This nonlinear PV pattern could be attributed to the decomposition of hydroperoxide into secondary oxidation products at high temperatures and with repeated high-temperature heating cycles [29] and has been reported in previous studies [30,31].

The IV of all experimental oils showed insignificant changes (*p* > 0.05) when heated up to 180 °C (Figure 3b), inferring that the level of unsaturation in the oil is not significantly affected by heating. A stable trend was also observed in the FFA content of coconut oil and palm olein upon heat exposure, except for palm olein at 180 °C, where a significant increase (*p* < 0.05) in FFA value was observed (Figure 3c), likely due to secondary oxidation products such as aldehydes, ketones, acids, alcohols, and hydrocarbons. The stable FFA trend in coconut oil and palm olein can be explained by their low moisture content as a result of refining activities. In contrast, the significantly higher (*p* < 0.05) FFA value in BSFL oil across increased heating, when compared with the control, is somewhat expected, as refining was not performed on this oil in the present study. The research of Mai et al. [5] reported a moisture content of 1.23% and 0.19% (85% reduction) for crude and refined BSFL oil, respectively, with reported FFA content of 5.97% for crude and 0.45% for refined BSFL oils (92% reduction). Overall findings suggest that the chemical properties of BSFL oil undergo prominent changes in PV and FFA, notably at the higher temperature of 180 °C.

### 3.4. Changes in the Perceived Odour Intensity of BSFL Oils With and Without Heating

The odour profile of BSFL oil was evaluated in two stages: (i) development of perceived odour descriptors and (ii) rating of odour intensity. A plethora of odour descriptors: “almond”, “cold meat fat”, “fish skin”, “fried oil”, “pungent”, “roast nut”, “salted egg”, et cetera, was frequently mentioned by the recruited panellist during initial odour attribute development (Supplementary Table S1). Ultimately, six dominant odour descriptors were agreed upon: “fishy”, “nutty”, “oily”, “meaty/savoury”, “roasted”, and “pungent” (for reference food item used, see Supplementary Table S2). These chosen odour descriptors aligned with those that have been used in literature to characterise the odour of insect-derived oils [2,11]. Subsequently, 10 trained panellists rated the intensity of these six odour attributes based on the definitions established during the training sessions (Table 3).

The mean intensity ratings for the perceived odour attributes of BSFL oil before and after heat treatments are presented in Table 4 and visualised in Supplementary Figure S5. In general, the odour intensity of all odour attributes increases as the heating condition increases. When compared with the control (unheated), “fishy” and “oily” odour intensity was significant (*p* < 0.05) at 180 °C, whilst insignificant differences (*p* < 0.05) were noted for “meaty/savoury”, “roasted”, and “pungent” at either 120 °C or 180 °C. The increase in fishy odour at higher temperatures could be linked to the oxidation of PUFA such as linoleic acid [4]. Low levels of ketones generated from the oxidation of linolenic acid may also contribute to a nutty odour [32]. An increase in roasted and nutty odour in BSFL oil at 180 °C could also be due to the formation of pyrazine, which is typically more prevalent at higher temperatures [33]. Furthermore, the rise in meaty/savoury odour at 180 °C could be caused by Maillard reactions between proteins and aldehydes [34]. Off-odours, such as rancid and pungent, also increased after thermal exposure, attributed to the oxidation of unsaturated fatty acids and the subsequent formation of volatile secondary lipid oxidation products such as aldehydes, alcohols, and ketones [35,36]. Overall, the intensified fishy and oily odour note at 180 °C may present a sensory hurdle that could limit the broader commercial application of BSFL oil in mainstream culinary uses, particularly in recipes or food products where a neutral or mild flavour profile is preferred. Such pronounced notes may be perceived as off-putting by consumers unfamiliar with insect-based ingredients. The unique “meaty/savoury” odour could, however, be appealing for specialised food applications that require the enhancement of the umami taste characteristic.

## 4. Conclusions

In conclusion, BSFL oil was found to be abundant in palmitic fatty acids (SAFA), oleic fatty acids (MUFA), and linoleic fatty acids (PUFA), providing a balanced distribution of saturated, monounsaturated, and polyunsaturated fatty acids, which contributed to its thermal stability properties. Whilst refined vegetable oils demonstrated greater stability across physical and chemical qualities, crude BSFL oil exhibited limitations in high-temperature cooking applications, primarily due to its higher PV and FFA levels, especially at 180 °C. Cooking at high temperatures also intensified the odour attributes of fishy, nutty, oily, meaty/savoury, roasted, and pungent. Altogether, the moderate oxidative stability of BSFL oil suggests potential for low-heat applications. Antioxidant fortification may improve stability, enabling greater use in high-heat applications. Refining processes could strengthen the quality of BSFL oil for commercial use by removing moisture, undesirable odours, and impurities.

Future research should focus on refining processing techniques of BSFL oil, conducting in-depth chemical analysis (including volatile compound profiling), and exploring culinary applications. In addition, sensory evaluation should be extended to a broader age demographic to improve generalisability. Further studies should also assess storage stability, as well as the toxicological and mutagenic safety of BSFL oil, to establish its viability as a safe and sustainable alternative to conventional cooking oils.

## Figures and Tables

**Table 1:** Fatty acid composition of BSFL oil, coconut oil, and palm olein before heating.

Carbon No.	Fatty Acid	BSFL Oil	Coconut Oil	Palm Olein	One-Way ANOVA Result
Saturated Fatty Acids (SAFA)					
C6:0	Caproic	ND	0.92 ± 0.01	ND	-
C8:0	Caprylic	ND	9.05 ± 0.01	ND	-
C10:0	Capric	0.51 ± 0.01	8.09 ± 0.01	ND	-
C12:0	Lauric	9.66 ± 0.04 ^b^	24.88 ± 0.21 ^a^	0.61 ± 0.01 ^c^	F_(2,6)_ = 29,058, *p* = 0.001
C14:0	Myristic	4.90 ± 0.02 ^b^	18.77 ± 0.11 ^a^	1.80 ± 0.03 ^c^	F_(2,6)_ = 56,103, *p* = 0.001
C16:0	Palmitic	23.69 ± 0.07 ^b^	15.09 ± 0.04 ^c^	30.24 ± 0.06 ^a^	F_(2,6)_ = 53,014, *p* = 0.001
C18:0	Stearic	4.46 ± 0.01 ^c^	5.90 ± 0.01 ^b^	6.51 ± 0.04 ^a^	F_(2,6)_ = 6276, *p* = 0.001
C20:0	Arachidic	ND	ND	0.59 ± 0.01	-
ΣSAFA	NA	43.22	82.70	39.75	-
Monounsaturated Fatty Acids (MUFA)					
C14:1	Myristoleic	0.60 ± 0.01	ND	ND	-
C16:1	Palmitoleic	3.46 ± 0.02	ND	ND	-
C18:1n9c	Oleic	30.90 ± 0.06 ^b^	13.97 ± 0.04 ^c^	45.02 ± 0.01 ^a^	F_(2,6)_ = 381,447, *p* = 0.001
Polyunsaturated Fatty Acids (PUFA)					
C18:2n6c	Linoleic	21.81 ± 0.11 ^a^	3.32 ± 0.04 ^c^	15.22 ± 0.03 ^b^	F_(2,6)_ = 59,282, *p* = 0.001
Total Unsaturated Fatty Acids (TUFA)					
ΣMUFA	NA	34.96	13.97	45.02	-
ΣPUFA	NA	21.81	3.32	15.22	-
ΣTUFA	NA	56.77	17.29	60.24	-

Values are mean ± standard deviation (*n* = 3) expressed as %. Different superscript lower-case letters within the same row indicate a significant difference (*p* < 0.05). ND denotes not detected. NA denotes not applicable. Σ denotes sum. ΣTUFA is calculated from the sum of MUPA and PUFA. ANOVA results are not provided for fatty acids that were not detected in one or both oil samples, as well as for the summary of total saturated and unsaturated fatty acids; these are therefore denoted as “-”.

**Table 2:** Physicochemical properties of BSFL oil, coconut oil, and palm olein before heating.

Properties	BSFL Oil	Coconut Oil	Palm Olein	One-Way ANOVA Result
Physical				
L*	36.00 ± 0.25 ^b^	69.80 ± 0.01 ^a^	69.20 ± 0.20 ^a^	F_(2,6)_ = 11,225, *p* = 0.001
a*	31.40 ± 0.40 ^a^	−0.80 ± 0.10 ^b^	−5.23 ± 0.32 ^c^	F_(2,6)_ = 13,162, *p* = 0.001
b*	57.23 ± 1.05 ^a^	14.30 ± 0.10 ^c^	36.00 ± 0.10 ^b^	F_(2,6)_ = 3692, *p* = 0.001
Viscosity (mPa•s)	72.00 ± 3.65 ^a^	53.00 ± 1.25 ^b^	67.93 ± 1.53 ^a^	F_(2,6)_ = 52, *p* = 0.001
Chemical				
Peroxide Value (meq/kg oil)	2.62 ± 0.14 ^b^	0.78 ± 0.20 ^c^	8.38 ± 0.51 ^a^	F_(2,6)_ = 448, *p* = 0.001
Iodine Value (g I_2_/100 g oil)	52.08 ± 1.25 ^b^	12.02 ± 0.71 ^c^	63.31 ± 0.82 ^a^	F_(2,6)_ = 2403, *p* = 0.001
Free Fatty Acid Value (%)	1.13 ± 0.03 ^a^	0.13 ± 0.01 ^b^	0.13 ± 0.01 ^b^	F_(2,6)_ = 3231, *p* = 0.001

Values are mean ± standard deviation (*n* = 3). Different superscript lower-case letters within the same row indicate a significant difference (*p* < 0.05). Colour indexes: L*, lightness (100 = white, 0 = black); a* (−*a* = greenness, +*a* = redness); b* (−*b* = blueness, +*b* = yellowness). Free fatty acid value is expressed as % oleic acid (for BSFL and palm olein) and % lauric acid (for coconut oil).

**Table 3:** Definition of odour attributes for the evaluation of BSFL oil.

Odour Attribute	Definition
Fishy	Perception of odour associated with the fishiness of any seafood product.
Nutty	Perception of odour associated with a mix of cashew nut and almond.
Oily	Perception of odour associated with rancid vegetable oil.
Meaty/savoury	Perception of odour associated with roasted or deep-fried meat with its crispy cracklings.
Roasted	Perception of odour associated with any roasted food product.
Pungent	Perception of odour associated with a noticeable odour that can be somewhat irritating.

These odour attributes were discussed and agreed upon by 10 trained sensory panellists.

**Table 4:** Intensity ratings for the perceived odour attributes of BSFL oil before and after heating.

Odour Attribute	Control	120 °C	180 °C	One-Way ANOVA Result
Fishy	75.47 ± 17.22 ^b^	98.98 ± 29.47 ^a^	96.09 ± 26.57 ^ab^	F_(2,18)_ = 3.79, *p* = 0.028
Nutty	85.18 ± 27.26 ^a^	73.40 ± 25.66 ^a^	93.55 ± 27.21 ^a^	F_(2,18)_ = 2.01, *p* = 0.143
Oily	71.81 ± 23.82 ^b^	85.80 ± 27.79 ^ab^	97.85 ± 20.14 ^a^	F_(2,18)_ = 4.27, *p* = 0.019
Meaty/Savoury	71.05 ± 19.43 ^a^	78.23 ± 17.66 ^a^	88.90 ± 18.18 ^a^	F_(2,18)_ = 2.16, *p* = 0.124
Roasted	77.49 ± 25.58 ^a^	71.11 ± 26.13 ^a^	92.81 ± 23.52 ^a^	F_(2,18)_ = 2.06, *p* = 0.137
Pungent	44.56 ± 22.68 ^a^	52.38 ± 36.89 ^a^	59.46 ± 32.52 ^a^	F_(2,18)_ = 0.81, *p* = 0.451

Values are mean ± standard deviation (*n* = 20) from 10 trained panellists who have evaluated BSFL oil samples before and after heating four times over two separate sessions. Different superscript lower-case letters within the same row indicate a significant difference (*p* < 0.05).

## Data Availability

The original contributions presented in the study are included in the article/Supplementary Materials. Further inquiries can be directed to the corresponding author.

## Supplementary Materials

The following supporting information can be downloaded at: https://www.mdpi.com/article/10.3390/foods14132333/s1, Supplementary Data S1: BSFL reading and oil extraction (Supplementary Figure S1. BSFL rearing and oil extraction); Supplementary Data S2: Fatty acid composition determination; Supplementary Data S3: Colour and viscosity determination; Supplementary Data S4: Peroxide value determination; Supplementary Data S5: Iodine value determination; Supplementary Data S6: Free fatty acid value determination; Supplementary Data S7: Odour profiling of BSFL oil using trained panellist (Supplementary Table S1: List of odour descriptors for BSFL oil generated during initial odour attribute development, Supplementary Table S2: Sensory descriptors, definitions and reference food items for odour profiling of BSFL oil); Supplementary Data S8: Standard for PV, IV, and FFA (Supplementary Table S3: Standard for peroxide, iodine, and free fatty acid values of refined coconut oil and palm olein); Supplementary Data S9: Representative GC-MS chromatograms of oils before and after exposure to heat (controls) (Supplementary Figure S2. GC-MS chromatograms); Supplementary Data S10: Stacked bar chart of fatty acid composition (Supplementary Figure S3. Fatty acid composition of BSFL oil, coconut oil, and palm olein); Supplementary Data S11: Overall appearance of BSFL oil after exposure to heat (Supplementary Figure S4. Colour variation of BSFL oil following exposure to 120 and 180 °C, illustrating progressive changes in visual appearance associated with thermal treatment); Supplementary Data S12: Spider plot of perceived odour intensity (Supplementary Figure S5. Mean perceived intensity for six key odour attributes for BSFL oil samples by 10 trained panellists across two independent odour evaluation sessions); Supplementary Data S13: Summary of findings (Supplementary Table S4: Summary of physicochemical properties and fatty acid composition for control/unheated BSFL oil (BO), coconut oil (CO) and palm olein (PO), Supplementary Table S5: Summary of physicochemical properties for heated BSFL oil (BO), coconut oil (CO) and palm olein (PO) as a factor of temperature, Supplementary Table S6: Summary of physicochemical properties for control/unheated (UH) vs. heated BSFL oil (BO), coconut oil (CO) and palm olein (PO)).
