# Supplementary material for: Physicochemical and Perceived Olfactory Changes in Black Soldier Fly (Hermetia illucens) Larvae Oil Under Domestic Cooking Temperatures
Source: Foods. 2025 Jun 30;14(13):2333. doi: 10.3390/foods14132333 (PMC12249279; doi:10.3390/foods14132333)
Supplement: Supplementary file 1 [file foods-14-02333-s001.zip › foods-3701493-supplementary.pdf]

Supplementary Material

Physicochemical and perceived olfactory changes in black soldier fly (*Hermetia illucens*) larvae oil under domestic cooking temperatures

Kian Aun Chang [1], Sze Ying Leong [1, 2*], Lye Yee Chew [1,2], Ching Qi Lim [1], Meng Jack Lim [1], Zongwei Ong [1], Sook Wah Chan [1, 2]

[1]School of Biosciences, Faculty of Health and Medical Sciences, Taylor's University, Subang Jaya 47500, Selangor Darul Ehsan, Malaysia
[2]Food Security and Nutrition Impact Lab, Taylor's University, Subang Jaya 47500, Selangor Darul Ehsan, Malaysia
*Corresponding author: szeying.leong@taylors.edu.my

Data S1. BSFL rearing and oil extraction

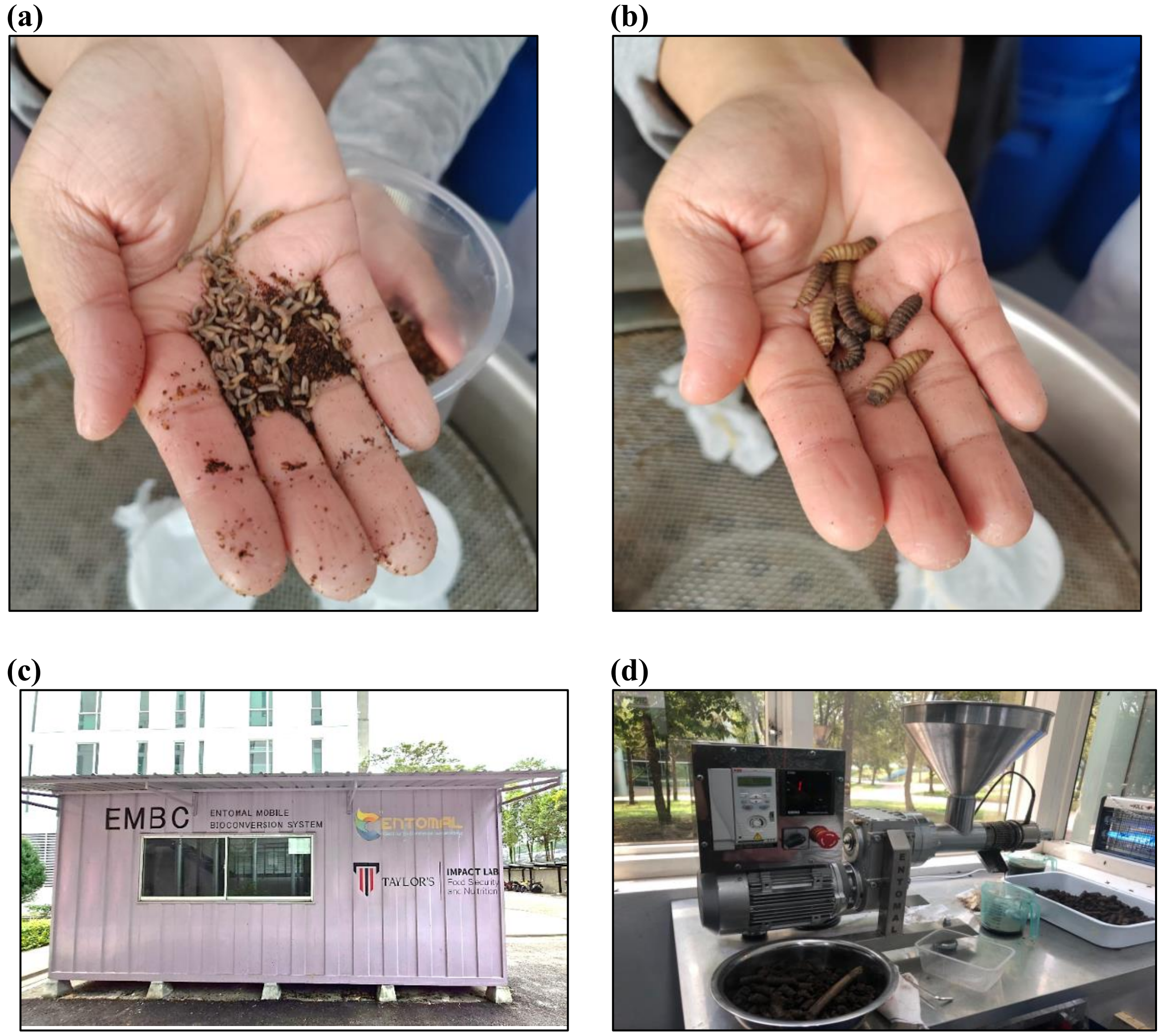

Supplementary Figure S1. BSFL rearing and oil extraction (a) Seven-day-old black soldier fly larvae (BSFL) before exposure to food waste, **(b)** BSFL after seven days of exposure to food waste; ready for harvesting, **(c)** mobile bioconversion system; where BSFL rearing takes place, and **(d)** hot-press machine used for BSFL oil extraction.

Data S2. Fatty acid composition determination

Fatty acids in the oil samples were converted into fatty acid methyl esters (FAMEs) following the International Union of Pure and Applied Chemistry (IUPAC) Commission on Oils, Fats and Derivatives Method 2.301 with minor modifications [17]. Approximately 100 mg of oil sample was mixed with 10 mL of hexane and 200 µL of 2N potassium hydroxide in methanol. The mixture was vortexed for 30 sec to ensure thorough mixing, then centrifuged at 4,000 rpm at 15 °C for 10 min. After centrifugation, the clear supernatant was filtered and carefully transferred to a gas chromatography (GC) glass vial for injection. The gas chromatography-mass spectrometry (GC-MS) parameters were optimised to enhance the separation and detection of FAMEs using Agilent 8990 GC system and Agilent 5977C GC/MSD unit (Agilent Technologies, Inc., California, USA). The column used was an HP-5MS UI with dimensions of 30 m × 250 µm × 0.25 µm (Agilent Technologies, Inc., California, USA), capable of handling temperatures ranging from –60 °C to 325 °C (with a maximum of 350 °C). The carrier gas was helium, with a flow rate of 1 mL/min, and the sample injection was performed at a volume of 1 µL in split mode with a split ratio of 20:1. The oven temperature programme began at 80 °C with a hold time of 4 min, increased to 220 °C at a rate of 25 °C/min within 4 min, and then to 250 °C at a rate of 5 °C/min with a hold time of 10 min. This resulted in a total run time of 29.6 min, followed by a post-run equilibration time of 1 min. Blank injections were performed before the first sample injection and three times after the final sample injection to ensure no carryover of residual components from previous runs. The standard used to determine the fatty acid composition was Supelco 37 Component FAME Mix (Merck KGaA, CRM47885, Darmstadt, Germany). The resulting chromatograms were analysed using Agilent MassHunter Workstation Software (Agilent, Santa Clara, CA, USA) to identify and quantify the fatty acids present in the samples. The fatty acid composition was expressed as the percentage of the peak area for each compound relative to the total peak area.

Data S3. Colour and viscosity determination

A pre-calibrated benchtop spectrophotometer (HunterLab, ColorFlex EZ, Murnau, Germany) was used to measure the colour of oil samples before and after stimulated heating conditions. The standard observer angle and illuminant were set at 10° and D-65, respectively. The oil samples were poured into a glass sample cup (64 mm in diameter) till they reached the indicator line and covered with an opaque cover before measurements were made. The colour indexes were expressed using $L*$, $a*$, and $b*$ tristimulus scale. The viscosity of the oil samples before and after heating was measured using a rotational viscometer (Brookfield, DV2T, Massachusetts, USA) with spindle LV-3 (63) at a constant speed of 200 rpm for 30 sec.

Data S4. Peroxide value determination

Peroxide value (PV) was determined following the Malaysian Palm Oil Board (MPOB) Test Method p2.3: 2004 [18]. Initially, 5 g of oil sample was added with 50 mL of acetic-isooctane solution (3:2, v/v). The solution was swirled till the sample had fully dissolved, and then 0.5 mL of saturated potassium iodide (KI) solution was added. The solution was allowed to stand for 1 min before being vigorously shaken for another 1 min. Next, 30 mL of deionised water was added, along with 0.5 mL of starch solution (0.5%, w/v), which acts as an indicator. The peroxide compounds present in the oil oxidised the iodide to iodine, which was then titrated against a standardised 0.01 N sodium thiosulphate solution until it turned from blue to colourless, indicating the endpoint. A blank titration was performed by repeating the same procedure without the oil samples. The amount of iodine produced was directly proportional to PV, which is expressed as milliequivalents of peroxide per kilogram of oil (meq/kg). The peroxide value (PV) of the oil samples was calculated using Equation (Eq.) S1:

$$\text{PV (meq/kg oil)} = [1000 \times (V_1 - V_0) \times N] / W \qquad \text{Eq. S1}$$

Where,
V_1 = volume of sodium thiosulphate solution used for titration of sample (mL)
V_0 = volume of sodium thiosulphate solution used for titration of blank (mL)
N = normality of sodium thiosulphate solution (determined using Eq. S2)
W = weight of oil sample (g)

S4.1 Standardisation of 0.01 N sodium thiosulphate for peroxide value calculation

The standardisation of 0.01 N sodium thiosulphate was conducted following the Malaysia Palm Oil Board (MPOB) Test Method p2.3: 2004. In this method, 0.01 g of potassium dichromate and 0.5 g of potassium iodide were measured in a 250 mL conical flask. Then, 10 mL of deionised water was added, and the solution was gently swirled until completely dissolved. Next, 10 mL of 10% hydrochloric acid solution was added and mixed thoroughly for 1 min. The solution was titrated against the prepared sodium thiosulphate until it turned light yellow, after which 1 mL of starch (0.5%, w/v) solution was added as an indicator. The titration was continued until the blue colour disappeared. This process was carried out in duplicate, and the normality (N) of sodium thiosulphate was calculated using Eq. S2:

$$\text{PV (N)} = (1000 \times K) / (49 \times T) \qquad \text{Eq. S2}$$

Where,
K = weight of potassium dichromate (g)
T = volume of sodium thiosulphate solution used (mL)

Data S5. Iodine value determination

Iodine value was determined following the MPOB Test Method p3.2:2004 [19]. Initially, 0.2 g of the oil sample was added with 20 mL of cyclohexane-acetic acid solution (1:1, v/v) and 25 mL of Wijs reagent. The mixture was gently swirled, covered, and left in the dark for 1 h. Next, 100 mL of deionised water and 20 mL of KI solution (10%, w/v) were added to convert any unreacted iodine monochloride into free iodine, resulting in a yellow-brown solution. The sample was then titrated against a standardised 0.1 N sodium thiosulphate solution, which reduces the free iodine to colourless iodide ions. The fading of the yellow colour during titration indicated a reduction in free iodine. Then, 0.5 mL of starch solution (1%, w/v) was added as an indicator, where the starch forms a blue complex with any remaining iodine. Titration continued until the blue colour disappeared, indicating that all free iodine had been reduced to iodide ions. A blank titration was performed by repeating the same procedure without the oil samples. The IV is expressed as g of iodine absorbed per 100 g of oil, representing the degree of unsaturation (number of double bonds) in the fatty acids of the oil sample. The iodine value of the oil samples was calculated using Eq. S3:

$$\text{IV (g } I_2\text{/100 g oil)} = [12.69 \times N \times (V_0 - V_1)] / W \qquad \text{Eq. S3}$$

Where,
N = normality of sodium thiosulphate solution (determined using Eq. S4)
V_0 = volume of sodium thiosulphate solution used for titration of blank (mL)
V_1 = volume of sodium thiosulphate solution used for titration of sample (mL)
W = weight of oil sample (g)

S5.1 Standardisation of 0.1 N sodium thiosulphate for iodine value analysis

The standardisation of 0.1 N sodium thiosulphate was performed according to the MPOB Test Method p3.2:2004. First, 25 mL of standard 0.1 N potassium dichromate was measured in a 250 mL conical flask. Then, 5 mL of concentrated hydrochloric acid and 10 mL of potassium iodide (10%, w/v) solution were added. The mixture was gently swirled and left to stand for 5

min. Afterwards, 100 mL of deionised water was added, and the solution was titrated against the prepared sodium thiosulphate until the yellow colour almost disappeared. Next, 1 mL of starch solution (1%, w/v) was added as an indicator, and titration was continued until the blue colour disappeared. This process was repeated in duplicate, and the normality (N) of sodium thiosulphate was calculated using Eq. S4:

$$\text{Sodium Thiosulphate (N)} = (25 \times N) / V \qquad \text{Eq.S4}$$

Where,

N = volume of sodium thiosulphate solution used (mL)

V = normality of standard potassium dichromate solution

Data S6. Free fatty acid value determination

Free fatty acid (FFA) value was determined according to the MPOB Test Method p2.5:2004 [18] with slight modifications. Firstly, 25 mL of isopropanol was heated to 40 °C, adding 0.25 mL of phenolphthalein solution, and titrating with 0.1 M sodium hydroxide solution until a permanent light pink colour was achieved. Next, 2.5 g of the oil sample was added with the neutralised isopropanol. The mixture was gently shaken and placed on a hot plate at 40 °C to allow the sample to fully dissolve. Then, 1 drop of phenolphthalein solution was added as an indicator, and the sample was titrated against a 5 mM sodium hydroxide (NaOH) solution until a light pink colour that lasted for at least 30 sec was observed. The FFA value was calculated based on the volume of NaOH used in the titration, where the amount of NaOH required to neutralise the free fatty acids was directly proportional to the FFA content in the oil sample. The free fatty acid (FFA) value was calculated using Eq. S5 and Eq. S6:

For BSFL oil and palm oil:

$$\text{FFA (\%) as oleic acid} = (28.2 \times N \times V) / W \qquad \text{Eq. S5}$$

Where,

N = normality of sodium hydroxide solution

V = volume of sodium hydroxide solution used (mL)

W = weight of oil sample (g)

For coconut oil:

$$\text{FFA (\%) as lauric acid} = (20.0 \times N \times V) / W \qquad \text{Eq. S6}$$

Where,

N = normality of sodium hydroxide solution

V = volume of sodium hydroxide solution used (mL)

W = weight of oil sample (g)

Data S7. Odour profiling of BSFL oil using trained panellist

S7.1 Panellist recruitment and training

Panellists were recruited through word of mouth and social media, resulting in the initial selection of 21 individuals (5 females, 16 males) aged 20 to 30. They were screened based on their non-smoking status, non-vegetarian diet, time availability, and their scores on the food neophobia scale (FNS). The rationale for using FNS scores as a panellist selection criterion was to ensure a balanced distribution of panellists with both low and high FNS scores. This approach aimed to capture a comprehensive range of both positive and negative odour descriptors associated with the BSFL oil samples. Additional sensory screening involved evaluating their sensitivity, acuity, and consistency in identifying odour stimuli during odour identification, ranking, and rating tasks conducted in Taylor's University Food Science Lab. Based on these criteria, 10 panellists were ultimately selected for the odour evaluation study of BSFL oil samples.

S7.2 Panellist training session

A total of 9 sessions (2 hours per session) consisting of 7 training sessions, 1 mock evaluation, and 1 review session on panellist performance were conducted. Vocabulary development took place during the first three sessions. The goal of these sessions was to develop a sensory vocabulary that accurately described and differentiated the odour profiles of the BSFL oil samples subjected to thermal treatments (control/unheated, 120 °C, 180 °C). During these sessions, panellists were exposed to all three BSFL oil samples and worked collaboratively to develop common descriptors for describing these samples (Supplementary Table S1). In the end, the panellists reached a consensus where six key odour attributes were agreed upon: fishy, nutty, oily, meaty/savoury, roasted, and pungent. In the following training sessions, a list of reference standards to define these attributes was developed concurrently (**Supplementary Table S2**). The panellists reached a consensus on what constituted each attribute, such as "fishy," "nutty," and "roasted" through group discussions and reference usage. Moreover, a standardised odour evaluation procedure was developed, defining how and when each odour attribute should be evaluated for the BSFL oil samples. During odour evaluation, each attribute was assessed by sniffing the headspace of the oil sample in amber bottles (without consumption), following the orthonasal olfaction pathway. Subsequent training sessions focused on the use of an unstructured line scale for rating attribute intensities. Following training, a mock evaluation was carried out to assess the ability of panellists to rate the perceived intensity of specific odour attributes across three BSFL oil samples. The performance of panellists was reviewed, and additional feedback and training were provided before the formal evaluation.

Supplementary Table S1

List of odour descriptors for BSFL oil generated during initial odour attribute development.

• Roasted insects	• Raw fish	• Paint
• Buttery	• Bleach	• Cracklings
• Fish skin	• Beach/ocean	• Malt
• Roast nut	• Solvent	• Deep fried meat
• Earth	• Mothball	• Fried oil
• Nut	• Chlorine	• Fish flavoured snack
• Cut grass	• Metallic	• Seafood
• Salted egg	• Plant root	• Roasted shell of nuts
• Grass	• Fish oil	• Dirt
• Roast pepper	• Prawn	• Prawn cracker
• Peanut	• Ammonia	• Burnt plastic
• Almond	• Rotten cold meat	• Butter cake
• Used oil	• Cold meat fat	• Savoury meat
• Pungent	• Roast meat	

Supplementary Table S2

Sensory descriptors, definitions and reference food items for odour profiling of BSFL oil.

Odour Descriptor	Definition	Reference food item
Fishy	Perception of odour associated with the fishiness of any seafood product.	Prawn cracker
Nutty	Perception of odour associated with a mix of cashew nut and almond.	Cashew nut and almond (1:1, w/w)
Oily	Perception of odour associated with rancid vegetable oil.	Virgin olive oil (exposed to light for more than 3 months)
Meaty/savoury	Perception of odour associated with roasted or deep-fried meat with its crispy cracklings.	Meat cracklings
Roasted	Perception of odour associated with any roasted food product.	Roasted coffee powder
Pungent	Perception of odour associated with a noticeable odour that can be somewhat irritating.	None

S7.3 Preparation of BSFL oil

BSFL oil samples were prepared by heating 80 mL of the oil at 120 °C and 180 °C for 30 min in a convection oven (Memmert, UF 110, Schwabach, Germany). After heating, 5 mL of each sample was transferred into 30 mL amber bottles with metal caps (Jargeous Sdn. Bhd., Kuala Lumpur, Malaysia) to allow volatile compounds to accumulate in the headspace. All samples were labelled with random three-digit codes and kept at a room temperature of 26 ± 2 °C for odour evaluation.

S7.4 Formal odour evaluation procedure of BSFL oil

During the odour evaluation, panellists were asked to take a deep sniff of the BSFL oil samples and rated the intensity of each odour attribute using an unstructured 150 mm line scale anchored at "None" and "Intense". Samples were presented in a randomised, balanced order to avoid bias. Between each sample, panellists neutralised their nasal passages by sniffing roasted coffee beans and took a 1 min break to prevent olfactory fatigue. Data was collected with EyeQuestion (Logic8 B.V., Gelderland, Netherlands).

S7.5 Odour evaluation ballot for BSFL oil

The sensory ballot was administered online using EyeQuestion software (Logic8 B.V., Gelderland, Netherlands). A 150 mm unstructured line scale was used, and panellists received training on how to use the line scale. During sample evaluation, panellists were asked to mark the perceived intensity of each odour attribute by placing an “X” along the scale. The software automatically measured the distance from the left anchor to the marked point and calculated the relative intensity based on the total scale length (150 mm). Reproducibility was verified to ensure the effectiveness of the training before the formal evaluation of BSFL oil.

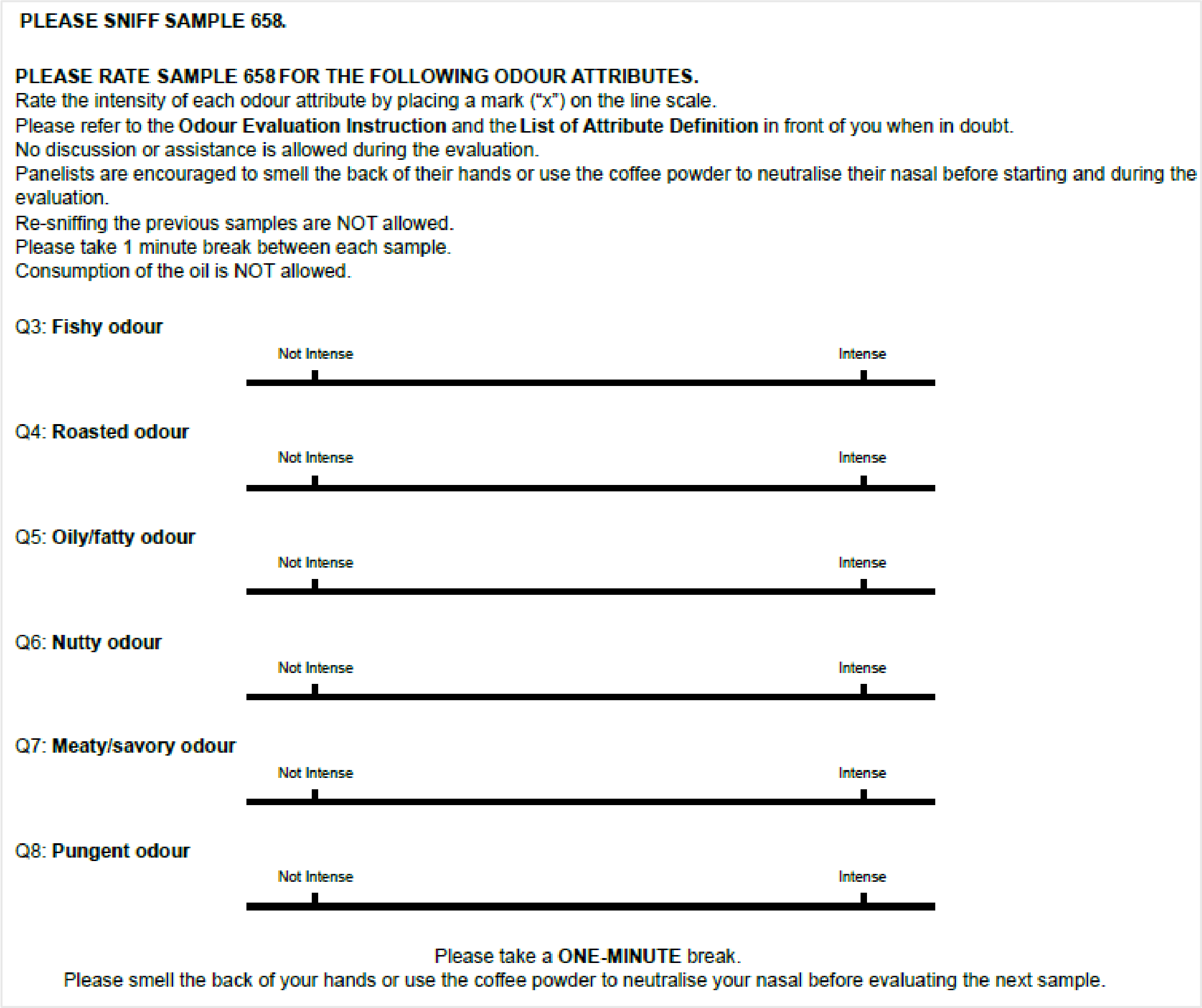

PLEASE SNIFF SAMPLE 658.

PLEASE RATE SAMPLE 658 FOR THE FOLLOWING ODOUR ATTRIBUTES.
Rate the intensity of each odour attribute by placing a mark (“x”) on the line scale.
Please refer to the **Odour Evaluation Instruction** and the **List of Attribute Definition** in front of you when in doubt.
No discussion or assistance is allowed during the evaluation.
Panelists are encouraged to smell the back of their hands or use the coffee powder to neutralise their nasal before starting and during the evaluation.
Re-sniffing the previous samples are NOT allowed.
Please take 1 minute break between each sample.
Consumption of the oil is NOT allowed.

Q3: **Fishy odour**
Not Intense — Intense

Q4: **Roasted odour**
Not Intense — Intense

Q5: **Oily/fatty odour**
Not Intense — Intense

Q6: **Nutty odour**
Not Intense — Intense

Q7: **Meaty/savory odour**
Not Intense — Intense

Q8: **Pungent odour**
Not Intense — Intense

Please take a **ONE-MINUTE** break.
Please smell the back of your hands or use the coffee powder to neutralise your nasal before evaluating the next sample.

Data S8. Standard for PV, IV, and FFA

Supplementary Table S3

Standard for peroxide, iodine, and free fatty acid values of refined coconut oil and palm olein.

Standard	Peroxide (meq/kg oil)		Iodine (g I_2/100 g oil)		Free Fatty Acid (%)	
	Coconut Oil	Palm Olein	Coconut Oil	Palm Olein	Coconut Oil	Palm Olein
Codex [24]	≤ 10.0[a]	≤ 10.0 [a]	6.3 – 10.6 [b]	≥ 56.0 [b]	–	–
Malaysia [26,27]	–	≤ 2.0	7.5 – 10.5	56.0 – 59.1	≤ 0.10	≤ 0.10

[a]Standard for refined vegetable oils; general.

[b]Standard for crude vegetable oils; specifically for coconut oil and palm olein.

Data S9. Representative GC-MS chromatograms of oils before exposure to heat (controls)

(a)

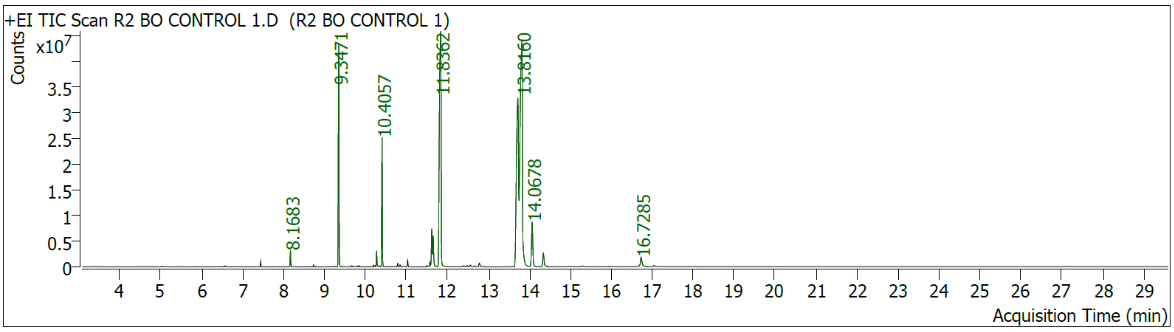

(b)

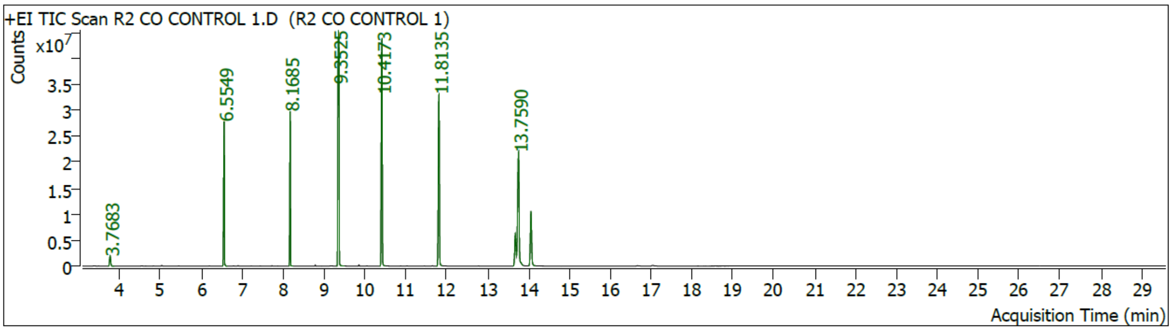

(c)

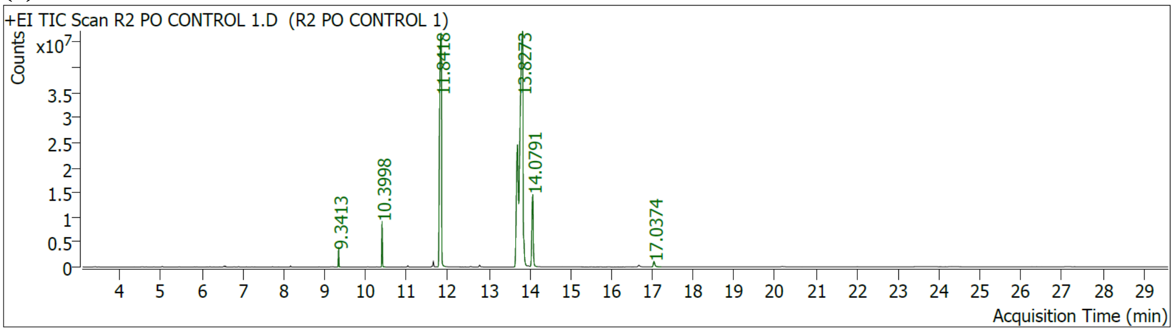

Supplementary Figure S2. GC-MS chromatograms (a) black soldier fly larvae (BSFL) oil; fatty acid and corresponding retention time: capric (8.1683 m), lauric (9.3471 m), myristoleic (10.2741 m), myristic (10.4057 m), palmitoleic (11.6531 m), palmitic (11.8362 m), linoleic (13.7187 m), oleic (13.8160 m), and stearic (14.0678 m) acids, **(b)** coconut oil; fatty acid and corresponding retention time: caproic (3.7683 m), caprylic (6.5549 m), capric (8.1685 m), lauric (9.3525 m), myristic (10.4173 m), palmitic (11.8135 m), linoleic (13.6789 m), oleic (13.7590 m), and stearic (14.0622 m) acids, **(c)** palm olein; fatty acid and corresponding retention time: lauric (9.3413 m), myristic (10.3998 m), palmitic (11.8418 m), linoleic (13.7072 m), oleic (13.8273 m), stearic (14.0791 m), and arachidic (17.0374 m) acids.

Data S10. Stacked bar chart of fatty acid composition

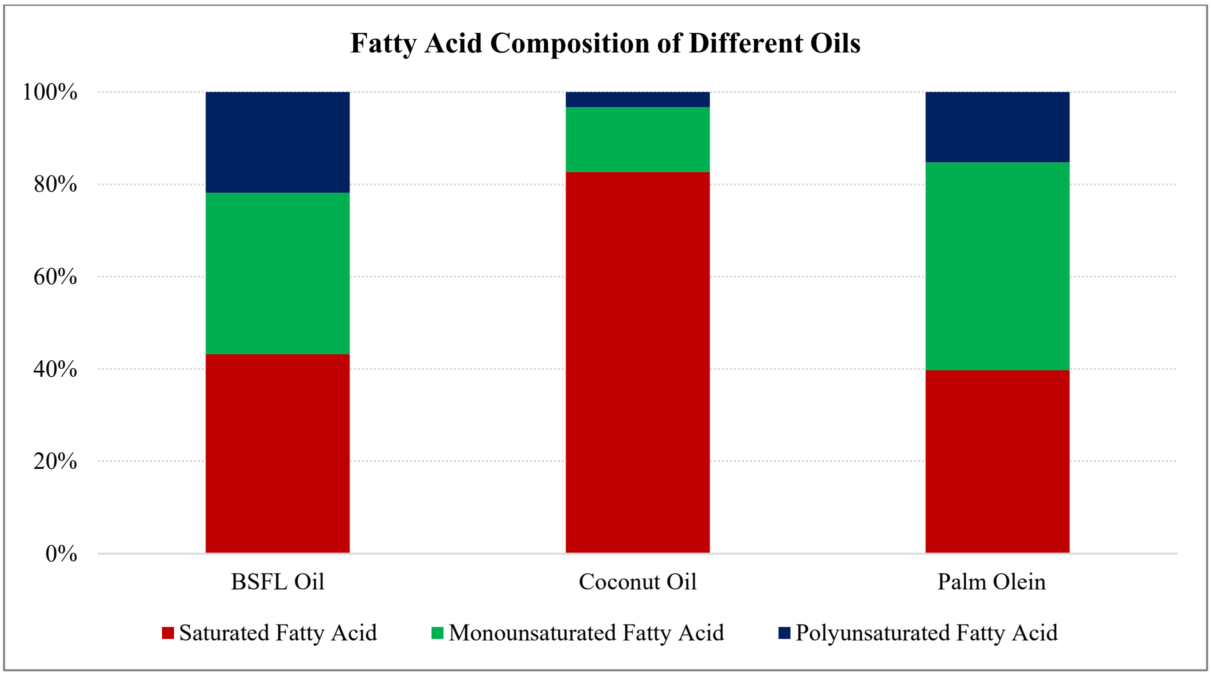

Supplementary Figure S3. Fatty acid composition of BSFL oil, coconut oil, and palm olein.

Data S11. Overall appearance of BSFL oil after exposure to heat

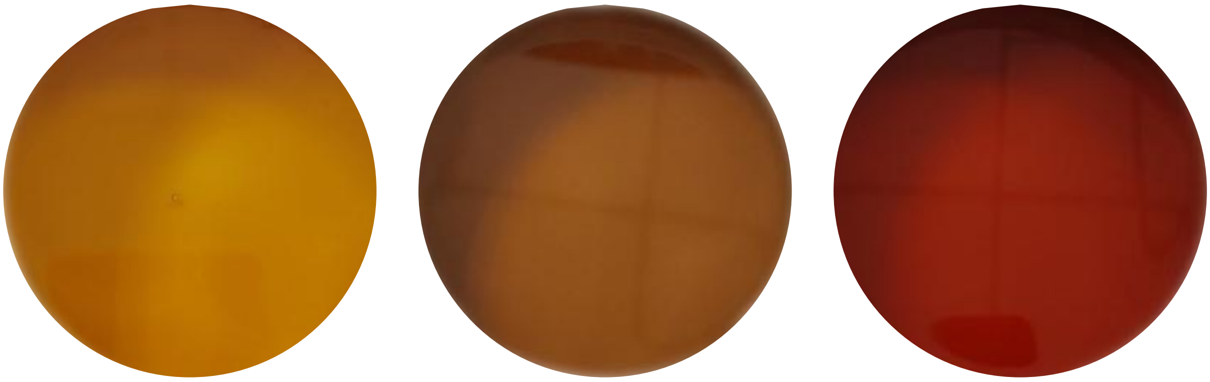

BSFL oil (unheated) BSFL oil (120 ºC) BSFL oil (180 ºC)

Supplementary Figure S4. Colour variation of BSFL oil following exposure to 120 and 180 ºC, illustrating progressive changes in visual appearance associated with thermal treatment.

Data S12. Spider plot of perceived odour intensity

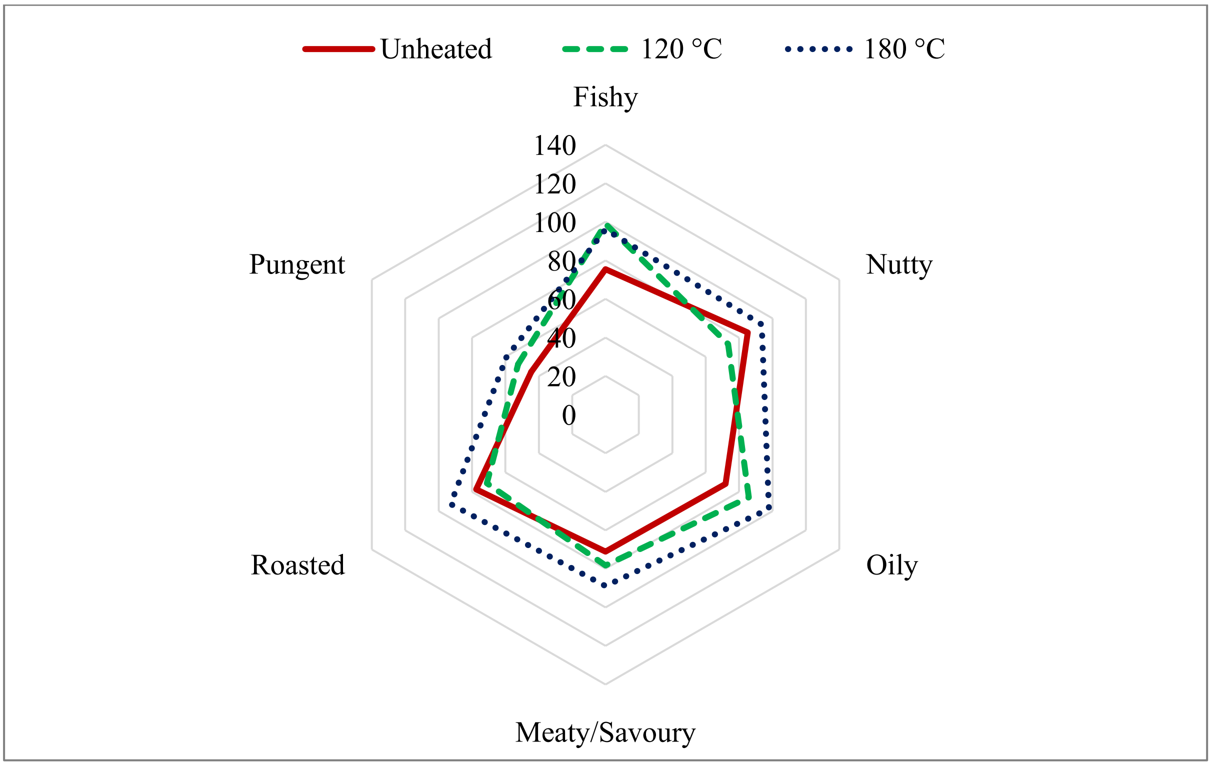

Supplementary Figure S5. Mean perceived intensity for six key odour attributes for BSFL oil samples by 10 trained panellists across two independent odour evaluation sessions.

Data S13. Summary of findings

Supplementary Table S4

Summary of physicochemical properties and fatty acid composition for control/unheated BSFL oil (BO), coconut oil (CO) and palm olein (PO).

Parameters	BO	BO vs. CO ($p < 0.05$)	BO vs. PO ($p < 0.05$)
L^*	Lower than CO & PO	✓	✓
a^*	Higher than CO & PO	✓	✓
b^*	Higher than CO & PO	✓	✓
Viscosity	Higher than CO & PO	✓	
PV	Higher than CO, lower than PO	✓	✓
IV	Higher than CO, lower than PO	✓	✓
FFA	Higher than CO & PO	✓	✓
ΣSAFA	Lower than CO, higher than PO	–	–
ΣMUFA	Higher than CO, lower than PO	–	–
ΣPUFA	Higher than CO & PO	–	–

<u>Trend Overview</u>

- Unheated BO is significantly darker, redder, and yellower than CO and PO.
- Unheated BO is significantly more viscous than CO.
- Unheated BO PV and IV are significantly higher than CO but lower than PO.
- Unheated BO FFA is significantly higher than CO and PO.
- Unheated CO has the highest ΣSAFA content when compared with CO and PO.
- Unheated PO has the highest ΣMUFA content when compared with CO and PO.
- Unheated BO has the highest ΣPUFA content when compared with CO and PO.

Symbol (✓) denotes there is a significant difference between different oils (BSFL Oil - BO, Coconut Oil - CO, Palm Olein - PO) at the same temperature (control/unheated). Symbol (–) denotes not applicable.

Supplementary Table S5

Summary of physicochemical properties for heated BSFL oil (BO), coconut oil (CO) and palm olein (PO) as a factor of temperature.

Parameters	120 °C			180 °C		
	BO	BO vs. CO ($p < 0.05$)	BO vs. PO ($p < 0.05$)	BO	BO vs. CO ($p < 0.05$)	BO vs. PO ($p < 0.05$)
*L**	Lower than CO & PO	✓	✓	Lower than CO & PO	✓	✓
*a**	Higher than CO & PO	✓		Higher than CO & PO	✓	✓
*b**	Higher than CO & PO	✓	✓	Higher than CO, lower than PO		✓
Viscosity	Higher than CO & PO	✓	✓	Higher than CO & PO	✓	✓
PV	Higher than CO, lower than PO		✓	Higher than CO, lower than PO	✓	✓
IV	Higher than CO, lower than PO	✓	✓	Higher than CO, lower than PO	✓	✓
FFA	Higher than CO & PO	✓	✓	Higher than CO & PO	✓	✓

Trend Overview

- BO heated at 120 °C and 180 °C is significantly darker than CO and PO heated at the same temperatures.
- BO heated at 180 °C is significantly redder than CO and PO heated at the same temperature.
- BO heated at 120 °C is significantly yellower than CO and PO at the same temperature.
- BO heated at 120 °C and 180 °C is significantly more viscous than CO and PO at the same temperatures.
- BO heated at 180 °C has significantly higher PV than CO and PO at the same temperature.
- BO heated at 120 °C and 180 °C has significantly higher IV than CO and PO at the same temperature.
- BO heated at 120 °C and 180 °C has significantly higher FFA than CO and PO at the same temperature.

Symbol (✓) denotes there is a significant difference between different oils (BSFL Oil - BO, Coconut Oil - CO, Palm Olein - PO) at the same temperature.

Supplementary Table S6

Summary of physicochemical properties for control/unheated (UH) vs. heated BSFL oil (BO), coconut oil (CO) and palm olein (PO).

Parameters	BO			CO			PO		
	UH	UH vs. 120 °C ($p < 0.05$)	UH vs. 180 °C ($p < 0.05$)	UH	UH vs. 120 °C ($p < 0.05$)	UH vs. 180 °C ($p < 0.05$)	UH	UH vs. 120 °C ($p < 0.05$)	UH vs. 180 °C ($p < 0.05$)
*L**	Lower than 120 °C, higher than 180 °C	✓	✓	Lower than 120 °C, higher than 180 °C	✓		Higher than 120 °C & 180 °C	✓	✓
*a**	Lower than 120 °C, higher than 180 °C		✓	Lower than 180 °C		✓	Lower than 120 °C & 180 °C	✓	✓
*b**	Lower than 120 °C, higher than 180 °C	✓	✓	Lower than 120 °C & 180 °C	✓	✓	Lower than 120 °C & 180 °C	✓	✓
Viscosity	Lower than 120 °C & 180 °C	✓	✓	Higher than 120 °C & 180 °C	✓	✓	Higher than 120 °C & 180 °C		
PV	Lower than 120 °C & 180 °C		✓	Lower than 120 °C & 180 °C	✓	✓	Lower than 120 °C & 180 °C	✓	✓
IV	Lower than 120 °C & 180 °C			Higher than 120 °C & 180 °C			Higher than 120 °C & 180 °C		
FFA	Lower than 120 °C & 180 °C	✓	✓	Lower than 120 °C & 180 °C			Lower than 120 °C & 180 °C		✓

Trend Overview

- For BO, heating at 120°C and 180 °C could cause a significant change in lightness, yellowness, viscosity, and FFA when compared with unheated BO.
- For CO, heating at 120°C and 180 °C could cause a significant change to yellowness, viscosity, and PV when compared with unheated CO.
- For PO, heating at 120°C and 180 °C could cause a significant change to lightness, redness, yellowness, and PV when compared with unheated PO.

Symbol (✓) denotes there is a significant difference between same oil (BSFL Oil - BO, Coconut Oil - CO, Palm Olein - PO) at different temperatures. UH denotes unheated (control).

References

Please refer to references [17], [18], [19], [24], [26], and [27] available in the main article.
